# Role of Different Subpopulations of CD8^+^ T Cells during HIV Exposure and Infection

**DOI:** 10.3389/fimmu.2017.00936

**Published:** 2017-08-07

**Authors:** Sandra Milena Gonzalez, Natalia Andrea Taborda, María Teresa Rugeles

**Affiliations:** ^1^Grupo Inmunovirología, Facultad de Medicina, Universidad de Antioquia UdeA, Medellín, Colombia; ^2^Grupo de Investigaciones Biomédicas Uniremington, Programa de Medicina, Facultad de Ciencias de la Salud, Corporación Universitaria Remington, Medellín, Colombia

**Keywords:** CD8^+^ T cells subpopulations, HIV infection, natural resistance to HIV, spontaneous control of HIV replication, antiviral immune response

## Abstract

During HIV infection, specific responses exhibited by CD8^+^ T cells are crucial to establish an early, effective, and sustained viral control, preventing severe immune alterations and organ dysfunction. Several CD8^+^ T cells subsets have been identified, exhibiting differences in terms of activation, functional profile, and ability to limit HIV replication. Some of the most important CD8^+^ T cells subsets associated with viral control, production of potent antiviral molecules, and strong polyfunctional responses include Th1-like cytokine pattern and Tc17 cells. In addition, the expression of specific activation markers has been also associated with a more effective response of CD8^+^ T cells, as evidenced in HLA-DR^+^ CD38^−^ cells. CD8^+^ T cells in both, peripheral blood and gut mucosa, are particularly important in individuals with a resistant phenotype, including HIV-exposed seronegative individuals (HESNs), long-term non-progressors (LTNPs) and HIV-controllers. Although the role of CD8^+^ T cells has been extensively explored in the context of an established HIV-1 infection, the presence of HIV-specific cells with effector abilities and a defined functional profile in HESNs, remain poorly understood. Here, we reviewed studies carried out on different subpopulations of CD8^+^ T cells in relation with natural resistance to HIV infection and progression.

## Introduction

HIV infection is currently one of the most important health problems worldwide; although continuous exposure to the virus may result in infection, an interesting group of repeatedly exposed individuals known as HIV-exposed seronegative individuals (HESNs), who do not exhibit any clinical or serological evidence of HIV infection, has been described (1, 2). Furthermore, among those who acquire the infection, different patterns of AIDS progression are observed: most infected patients progress to AIDS after 8–10 years, but some uncommon patients, known as long-term non-progressors (LTNPs), remain asymptomatic for more than 10 years, exhibiting low viral loads in the absence of antiretroviral therapy (3). In addition, the HIV-controllers exhibit a sustained and spontaneous control of the viral replication for at least 1 year after diagnosis with viral loads below 2,000 copies/mL (4).

Some genetic and immunological mechanisms have been associated with the resistant phenotype as follows: (i) genetic polymorphisms in the required co-receptors for viral entry, such as CCR5 or CCR2 (5, 6); (ii) increased production of the co-receptor ligands MIP-1α/β, RANTES or SDF-1; (iii) the presence of antibodies blocking viral co-receptors (7); (iv) the expression of different microRNAs induced by the viral exposure (8, 9) that may modulate the innate immune system or interfere directly with viral mRNAs blocking the infection (10); (v) induction of spontaneous apoptosis of target cells (6, 11); (vi) production of soluble factors with antiviral activity, such as TRIM5α, APOBEC-3G, SAMHD-1, serpina1, elafin, Human neutrophil peptide, beta-defensins, and LL-37 (12–14), other proteins implicated in host defense and bacterial binding, such as bPRP2, Histatin-3, Lysozyme C, and SLPI (15), and the presence of non-cationic proteins in genital secretions with HIV-1 neutralizing activity (16); (vii) high activity of natural killer cells (17, 18) and dendritic cells (DC) (19); (viii) high levels of neutralizing IgA antibodies (18, 20), which are even associated with protection in vaccine clinical trials, as they could prevent HIV mucosal transcytosis (21); (ix) expression of the alleles HLA-B57 and -B27 that present immunodominant peptides (6); and (x) effective and polyfunctional profile of HIV-specific CD4^+^ and CD8^+^ T cell responses (22, 23).

Some of these mechanisms that are so far associated with the resistant phenotype have been extensively studied to determine the immune correlates of protection that may serve as therapeutic targets. In particular, the induction of CD8^+^ T cells seems to constitute a key element for a potential vaccine, as the cells directed against more conserved peptides are crucial for viral control (24). Indeed, in macaques, the vaccination with immunodominant epitopes of the simian immunodeficiency virus (SIV), which is closely related to HIV, induced a high frequency of SIV-specific CD8^+^ T cells able to control viral replication (25).

The immune response of CD8^+^ T cells is triggered since early stages of HIV infection, and a polyfunctional response has been associated with viral control. In fact, this response is related to the establishment of a low viral set point, which is considered a predictor of slower rate of disease progression in LTNP and HIV-controllers (26–28). However, much less is known regarding their importance during viral exposure in HESNs. In those individuals, the HIV-specific CD8^+^ T cells appear to be increased, exhibiting lower activation, higher effector abilities, and a specific functional profile, with IFN-γ secretion after HIV stimulation *in vitro* (29, 30).

Thus, the intriguing question is how to explain the presence of the HIV-specific CD8^+^ T cells in the absence of an established infection, as occurs in HESNs. During sexual transmission, this could be the result of an abortive primary infection after the virus enters the mucosal barrier, considering that at least early events of the viral replication cycle are required to present viral peptides in the context of class I MHC molecules to elicit a CD8^+^ T cell response. The systemic activation of a specific response could then be responsible for avoiding the establishment of the infection (31). Nonetheless, it could also be related to the presence of a heterologous T cell response to similar antigens (32).

Although several studies have reported the presence of such specific cells, the functional phenotype of the CD8^+^ T cells that are more efficient in avoiding the establishment of HIV infection and/or controlling viral replication remains to be clarified. To approach this question, we reviewed recently published studies carried out on different subpopulations of CD8^+^ T cells in relation to natural resistance to HIV infection and progression.

## Role and Importance of CD8^+^ T Cells During the Antiviral Response

CD8^+^ T cells are a subpopulation of T cells that have a relevant role in host defense mainly against viruses and tumor cells. Effector cell differentiation occurs when naïve CD8^+^ T cells are activated by antigen-presenting cells (APCs), specifically DCs, that present endogenous peptides in the context of class I MHC molecules. In addition, they require the interaction with co-stimulatory molecules, such as CD80/86, and signaling through cytokines, usually provided by DCs and activated CD4^+^ T cells (33, 34); however, some studies have indicated little or no requirement for additional signaling coming from the CD4 compartment, at least under certain circumstances (35, 36).

Once naïve-specific CD8^+^ T cells are activated, the effective response requires clonal expansion and formation of primary effector cells capable of recognizing peptides from virally infected or tumor cells, leading to direct killing of antigen-bearing cells through perforins, granzymes, and Fas/FasL interaction (33, 37–39). In addition, release of cytokines with antimicrobial action, such as TNF-α and IFN-γ (40), and chemokines, such as MIP-1α/β and RANTES (41); all these mechanisms contribute to clearance of altered cells.

The concentration and antigen persistence play an important role in the differentiation into different subsets of T cells. Although a brief exposure to an antigen presented by APCs can trigger activation, expansion, and differentiation of naïve CD8^+^ T cells into effector T cells, prolonged exposure to the antigen is usually required to generate an efficient effector response and memory CD8^+^ T cells (33, 42). After resolution of an infection or a tumor process, a phase of T cell contraction is induced as a mechanism of immune regulation, during which most of the effector specific-CD8^+^ T cells die by apoptosis and some survivor cells (5–10%) are preserved as long-lived memory cells (33, 37).

Despite an effector response of CD8^+^ T cells, the successful eradication of the pathogen is not always guaranteed. In this sense, chronic infections such as HIV are characterized by antigen persistence that induces terminally differentiated effector CD8^+^ T cells over the memory phenotypes, and ultimately immune exhaustion and activation-induced cell death (43). In fact, late phases of HIV infection are associated with progressive reduction of CD8^+^ T cells, lower effector functions, and inability to respond to HIV and other pathogens or tumor cells (44–46).

## Functional Subsets of CD8^+^ T Cells

Once activated, the CD8^+^ T cells may differentiate into several functional phenotypes. Initially, they acquire an effector phenotype that will result in high numbers of terminally differentiated effector cells (85–90% of activated CD8^+^ T cells). This effector phenotype is characterized by high cytotoxic ability and production of cytokines; nonetheless, as the pattern of produced cytokines is variable among CD8^+^ T cells, they can be classified into different subsets of effector cells: (i) CD8^+^ T cells with a Th1-like cytokine pattern (Tc1), which produce IFN-γ and TNF-α and exhibit a high cytotoxic function and (ii) CD8^+^ T cells with a Th2-like cytokine pattern (Tc2), which produce IL-4, IL-5, IL-6, and IL-10 and have lower cytotoxic ability than Tc1 cells. In addition, some cells produce both Th1 and Th2 cytokines; interestingly, naïve CD8^+^ T cells exhibit a strong preference to differentiate into Tc1 cells (47). An additional pattern recently described and less studied is Tc17 that produces IL-17 but no granzyme B, and has a low capacity to perform lysis *in vitro*; these cells exhibit some functional plasticity, since they can produce IFN-γ while losing the expression of IL-17 (48).

Once the pathogen is eradicated, some cells acquire a memory phenotype that persists even in the absence of the antigen (49, 50). These memory CD8^+^ T cells are characterized by their capacity of self-renewal, reside in lymphoid and non-lymphoid tissues awaiting a second encounter with the specific antigen, and recall effector functions after this encounter (51). They are distinguished from naïve and effector CD8^+^ T cells that express the marker CD45RA, by the loss of this marker and the gain of CD45RO after differentiation (52). These memory CD8^+^ T cells have been classified into the subsets as follows, considering differences in the degrees of effector functions, proliferative capacity, and tissue homing properties: (i) central memory cells restricted mainly to lymphoid tissues because their expression of the lymphoid homing molecules CD62L and CCR7, which are also considered as the source for self-renewal of the pool of memory cells, generating a second wave of effector T cells; (ii) effector memory cells that provide a first line of defense against infections through immediate effector functions and are present in circulation and non-lymphoid tissues due to the low expression of the lymphoid homing molecules CD62L and CCR7 (37, 51); and (iii) a recently described subset of tissue-resident memory cells that are located at sites of pathogen entry. The protective CD8^+^ T cell response is achieved through the collective function of all these effector and memory subsets (51). A summary of effector and memory phenotype markers of CD8^+^ T cells is shown in Table 1.

**Table 1:** Subpopulations of CD8^+^ T cells according to effector and memory phenotype markers.

Phenotype	Surface-expressed markers
Naïve CD8^+^ T cells	CD45RA^+^ CD45RO^−^ CCR7^+^ CD62L^+^
Effector CD8^+^ T cells	CD45RA^+^ CD45RO^−^ CCR7^−^ CD62L^−^
Effector memory CD8^+^ T cells	CD45RA^−^ CD45RO^+^ CCR7^−^ CD62L^−^
Central memory CD8^+^ T cells	CD45RA^−^ CD45RO^+^ CCR7^+^ CD62L^+^

In addition to the effector and memory subsets, these CD8^+^ T cells can also be classified according to the level of activation; based on the expression of the activation markers HLA-DR and CD38, four phenotypes of cells have been identified: (i) HLA-DR^+^CD38^+^; (ii) HLA-DR^+^CD38^−^; (iii) HLA-DR^−^CD38^+^; and (iv) HLA-DR^−^CD38^−^.

The co-expression of HLA-DR and CD38 define the classical activation phenotype of CD8^+^ T cells. Several studies indicate that this population exhibits high effector functions, such as proliferation, cytotoxicity, and cytokine production, as well as higher susceptibility to cell death after their function have been accomplished (53, 54). Indeed, this subpopulation of activated specific CD8^+^ T cells performs an important function during acute viral infections, contributing to viral control (26); however, the maintenance of this activation state observed during chronic viral infections is related to the subsequent loss of their functional abilities, to increased expression of inhibitory molecules related to immune exhaustion, and to activation-induced cell death (55). Some studies have focused on the subpopulation HLA-DR^+^CD38^−^CD8^+^ T cells, particularly in the context of chronic viral infection (56, 57). This phenotype has been related to a controlled activated phenotype because of its low expression of the proliferation marker Ki-67 and of additional activation markers, such as CD69, CD25, CD71, and CD40. Indeed, the expression of these markers is similar to the one observed in resting HLA-DR^−^CD38^−^CD8^+^ T cells and lower than the one expressed by HLA-DR^+^CD38^+^CD8^+^ T cells. Remarkably, despite this lower activation, HLA-DR^+^CD38^−^CD8^+^ T cells exhibit a higher functional response, an increased survival rate and a greater ability to suppress viral replication compared to cells expressing both activation markers (56, 57). This particular activation phenotype seems to be induced by a higher avidity in the recognition of viral epitopes in the presence of low peptide concentrations (57).

In addition to these described populations, some CD8^+^ T cells exhibit the ability to suppress T helper activity and induce anergy, called regulatory CD8^+^ T cells (58–60); however, they are not well characterized. Some reports indicate that these cells are related to a memory phenotype since they are CD45RA negative; in addition, they express the CD122 marker but neither the CD25 nor the FoxP3 markers, and their regulatory function seems to be mainly achieved by IL-10 production. Although other mechanisms, such as cytotoxicity could also be involved in regulating effector cells, this issue requires further studies (61).

## HIV Pathogenesis and the Role of CD8^+^ T Cells During HIV Infection

HIV infection is characterized by massive depletion of activated CD4^+^ T cells in peripheral blood but mainly in the gut-associated lymphoid tissue (GALT); this cell elimination induces structural damage, loss of the mucosal integrity, and consequently microbial translocation from the intestinal lumen to systemic circulation (62). Subsequently, excessive and generalized immune activation of almost all immune cells is established. The immune hyperactivation initiates during early infection and is maintained throughout the chronic phase; it is currently accepted as the main pathogenic mechanism of HIV infection and is considered the best predictor of AIDS progression (63). This phenomenon contributes by increasing the number of viral target cells, augmenting the production of viral particles, and leading to quantitative and functional alterations of different components of the innate and adaptive immune responses (64).

The pathogenesis of the infection and the rate of HIV progression to AIDS is related to the viral control achieved as of the early stage of the infection, which is mainly mediated by CD8^+^ T cell responses, including both the cytotoxic and the non-cytotoxic antiviral response (65–67). In fact, an inverse correlation between specific responses of these cells and viral loads has been established. While the cytotoxic activity induces death of HIV-infected cells through granzymes, perforin and the Fas–FasL pathway, the antiviral molecules produced by CD8^+^ T cells inhibit viral entry or virus replication (68).

The magnitude and rapid onset of CD8^+^ T responses after acquiring the infection is crucial to determine viral control, since a high frequency and activation of HIV-specific CD8^+^ T cells appearing during the hyperacute infection are associated with a low viral set point, which is considered a predictor of slow disease progression (26, 27). In this respect, the establishment of a potent but controlled response of CD8^+^ T cells as of early infection is related to better control of the infection and delayed AIDS progression (26). In addition, the ability to maintain this response during the chronic phase is critical, as the depletion of CD8^+^ T cells in the SIV model showed a loss of viral control (69). Supporting the crucial role of CD8^+^ T cells in HIV, vaccine strategies in the SIV model promote the response of this population reducing viral replication once the challenge is established (70); in this respect, the use of some prospective vaccines tested in humans designed to trigger a strong response of this cell population showed promising results (71–73).

Other important aspect regarding CD8^+^ T cells response during HIV infection is the memory differentiation pattern; in infected patients, the most frequent subset is the pre-terminally differentiated CD45RA^−^CCR7^−^ cells, followed by CD45RA^+^CCR7^+^ cells, whereas the terminally differentiated CD45RA^−^CCR7^+^ cells are found in a lower frequency (74). The proliferative capacity of these subsets is variable, observing a more rapid division in precursor memory cells (CD45RA^+^CCR7^+^) after stimulus (74). Interestingly, the frequency of these subpopulations varies according to the phase of the infection, finding a higher proportion of central memory (CCR7^+^CD45RO^+^) and effector memory (CCR7^−^CD45RO^−^) CD8^+^ T cells during the acute stage; while in the chronic phase, there is a predominance of effector memory only (75). Furthermore, the differentiation and functional state of CD8^+^ T cells is also influenced by the expression of the costimulatory receptors CD28 and CD27, involved in regulating T cell activation. Among the three subsets so far described: CD28^+^CD27^+^, CD28^−^CD27^+^, and CD28^−^CD27, the intermediate subset CD28^−^CD27^+^, which exhibits lower proliferation and reduced cytotoxic potential (76), is found in higher frequency in HIV infected individuals (76).

**Figure 1:**
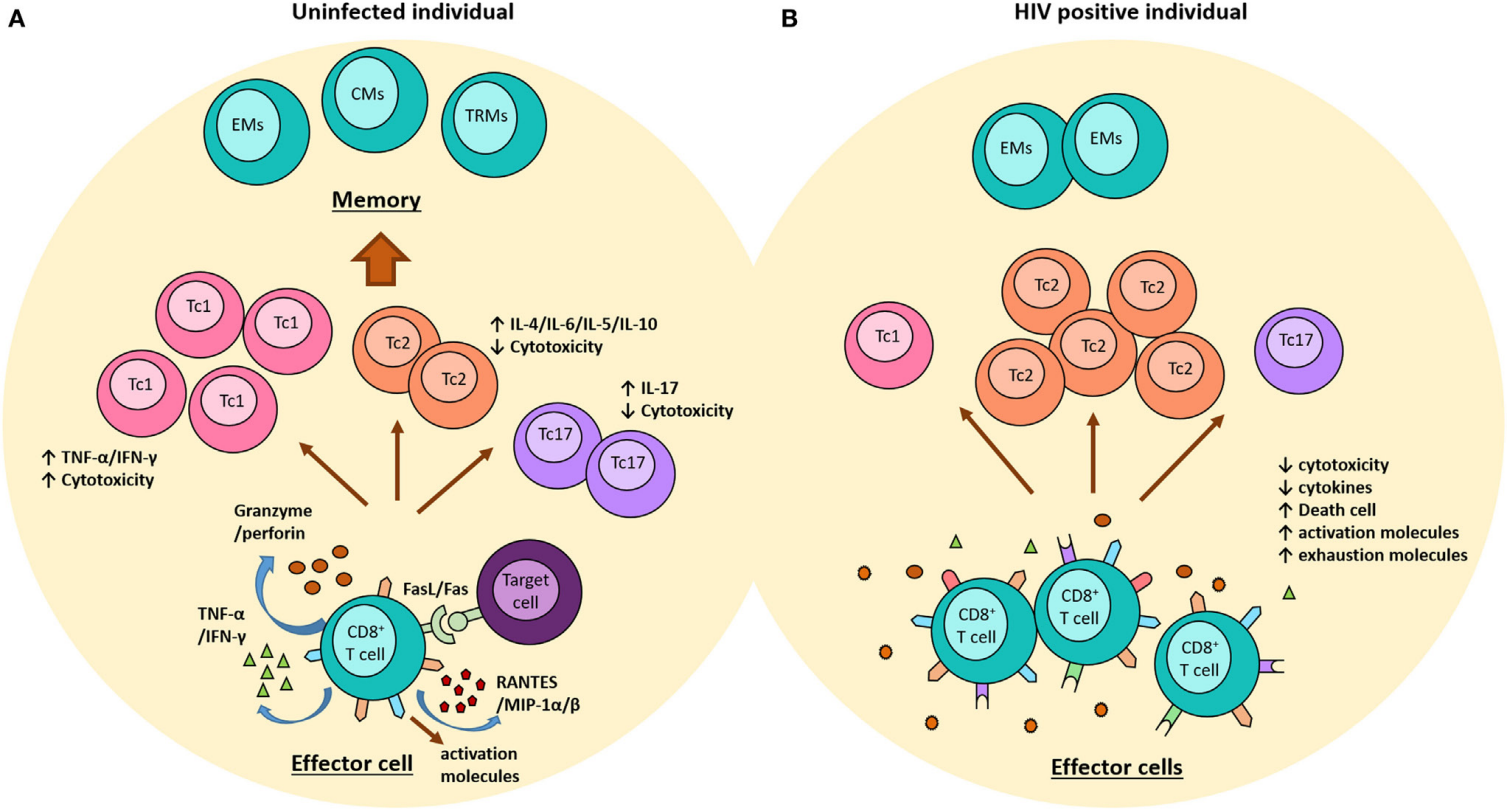
Role of CD8^+^ T cells in the context of the HIV-1 infection. **(A)** In uninfected individuals, once CD8^+^ T cells are activated, they acquire an effector phenotype characterized by high production of granzyme, perforin, and some pro-inflammatory molecules, such as RANTES and MIP-1α/β. In addition, these cells upregulate the expression of FAS-ligand. Some subpopulations of CD8^+^ T cells have been reported, including (i) Th1-like cytokine pattern (Tc1) cells have strong cytotoxic function and produce high levels of IFN-g and TNF-α; (ii) Th2-like cytokine pattern (Tc2) cells produce IL-4, IL-5, IL-6, and IL-10 and have lower cytotoxic ability; and (iii) Tc17 cells produce high levels of IL-17 but no granzyme. Once the pathogen is erradicated, the majority of effector cells die, and some become memory cells (EMs, effector memory cells; CMs, central memory cells, and TRMs, tissue resident memory cells). **(B)** During the chronic phase of the HIV-1 infection, the continuous viral replication induces the persistence of effector CD8^+^ T cells expressing high levels of activation markers, which progressively loss their functional activity and start expressing exhausting molecules, such as PD-1, CTLA-4, and Tim-3. This phenotype is characterized by low cytotoxicity and cytokines production. During this infection, there is a predominance of Tc2 cells and a reduction of Tc1 and Tc17. In addition, memory cells are decreased and there is a preferential differentiation into EMs.

Certainly, HIV infection is so complex that the potent suppressive response exerted by these cells to control viral infection might induce a strong selective pressure on the virus with consequent viral escape, increasing viral diversity, viral replication, and progression to AIDS, or conversely it might result in the loss of the viral fitness and better viral control (77, 78). Furthermore, the persistence of HIV infection and the excessive immune activation that is characteristic of the infection imply a constant challenge for these cells leading to progressive loss of their ability to respond to the antigens presented and the development of immune exhaustion (55, 79). In such a situation, HIV-specific CD8^+^ T cells exhibit increased expression of surface inhibitory molecules, such as PD-1, CTLA-4, or Tim-3, reduced production of cytokines and cytotoxic molecules, and an increased susceptibility to cell death (Figure 1) (80, 81). In addition, the response mediated by regulatory CD8^+^ T cells has been associated with advanced disease, as these cells suppress the cytolytic ability of HIV-specific CD8^+^ T cell, and also limit the production of IL-2 by direct cell–cell contact mechanisms (82).

## Role of CD8^+^ T Cells in Natural Resistance to HIV Infection

As just described, the response of CD8^+^ T cells has been reported to be one of the main mechanisms involved in controlling viral replication during HIV infection. In fact, a better response of these cells is frequently related to the HIV controller phenotype, since these individuals exhibit immunodominant HIV-specific CD8^+^ T cell responses in both periphery and GALT tissue, and a higher proliferative and cytotoxic capacity (56, 83, 84). In fact, HIV-controllers exhibit a higher functional response of specific CD8^+^ T cells, detecting cells reaching up to five simultaneous functions, including CD107a, IFN-γ, MIP-1β, IL-2, and TNF-α in response to Gag peptides; as expected, the higher frequency of these polyfunctional cells is inversely correlated with the viral load (85). Nonetheless, some of the HIV-controller individuals do present weak responses of these cells, suggesting that other mechanisms are also important in inducing and maintaining this controller phenotype (86). Despite this, the CD8^+^ T cell response remains the main mechanism of viral control described in the majority of HIV-controllers; in this respect, the phenotype of these cells in terms of effector and memory phenotypes and in level of activation could also have a different impact on viral control. In HIV-controllers, the effector memory and terminal effector subpopulations exhibit a high inhibitory potential to suppress the infection *in vitro*, and these subpopulations respond more rapidly to the infection (87). Similar results were observed in the SIV model, where vaccination inducing high numbers of effector memory CD8^+^ T cells led to the establishment of a controller phenotype (88).

By contrast, other reports indicate that these individuals have low or absent response of CD8^+^ T cells with an effector phenotype, maintaining highly functional central memory responses (89). In these individuals, a transitional memory phenotype of CD8^+^ T cells with non-cytotoxic antiviral responses, and exhibiting high HIV-suppressor activity were also described (90). However, other studies reported that this memory phenotype does not predict the functional ability of CD8^+^ T cells and that there was no over-representation of cells with a central memory phenotype in polyfunctional responses (85). In addition to the effector and memory phenotypes, some subpopulations of CD8^+^ T cells, defined according to the expression of activation markers, seem to play a role in the viral control exhibited by HIV-controllers. In these individuals, a low frequency of CD8^+^ T cells co-expressing HLA-DR and CD38, a subpopulation related to a less efficient control of infection, have been described compared to HIV-progressors by other researchers and by us (57, 83). In addition, controllers exhibited a higher frequency of CD8^+^ T cells expressing the activation marker HLA-DR but not CD38, mainly in HIV-specific cells; this activation phenotype was associated with better survival capacity, higher frequency of polyfunctional cells, and greater proliferative and cytotoxic capacity that results in higher ability to suppress the virus (56, 57). In fact, we observed a predominance of HLA-DR^+^CD38^−^ CD8^+^ T cells over the double-positive cells in HIV-controllers and, interestingly, it was related to a regulation profile and lower progression in terms of viral load and CD4 count (91). These results are consistent with other reports in which expression of CD38 on CD8^+^ T cells was related to high viral load and disease progression to AIDS (92, 93). Indeed, the expression of CD38 was induced *in vitro* by high concentrations of HIV peptides and also by the presence of pro-inflammatory cytokines, especially IFN-α, whereas the phenotype HLA-DR^+^CD38^−^ was induced by low concentrations of viral peptides as observed *in vivo* by HIV-controllers that maintain low viral loads (57). In HIV-controllers, we also observed an increased frequency of resting CD8^+^ T cells, HLA-DR^-^CD38^−^ compared to HIV-progressors (91). It has been suggested that the effective immune response to low viral load in HIV-controllers could be partly related to a higher avidity of CD8^+^ T cells to recognize the virus (94).

The Tc1, Tc2, and Tc17 subpopulations are also involved in the HIV response. In this regard, upon antigenic stimulation, Tc1 cells acquire a functional phenotype characterized by a high cytotoxic capacity through Fas–FasL interaction and degranulation of perforin and granzymes (95). Under physiological conditions, most CD8^+^ T cells exhibit a Tc1 profile induced by IL-12 and IFN-γ. However, during HIV infection, a poor production of IL-12 can induce polarization from Tc1 to Tc2 cells, decreasing the cytotoxic capacity against infected cells (96). In addition, Tc2 cells reduce the production of IL-2 by Tc1 cells, thus decrease cytokine production, proliferation, and cytotoxicity (97, 98). Interestingly, HIV patients exhibit a higher frequency of CD8^+^ T cells producing IL-4 than healthy individuals (19). Finally, Tc17 cells are particularly important during the HIV infection, because of their ability to inhibit microbial translocation into gut mucosa, by promoting the proliferation of enterocytes (99–102). However, HIV has drastic effects on the frequency and function of these cells, which are important in preserving CD4^+^ T cells in long-term non-progressor patients (103).

Although the main role of CD8^+^ T cells has been outlined once HIV infection is established, some studies reported the presence of HIV-specific CD8^+^ T cells in HESNs with high effector capacity, suggesting that these cells could also be implicated in avoiding the establishment of the infection and that they could be crucial for the development of a successful preventive vaccine (104–106). The presence of these cells could result from an abortive local infection of cells that are refractory to productive infection or have low levels of viral replication; it may occur during the eclipse phase when the infection can still be eradicated, eliminating the infected cells before viral dissemination (31, 107, 108).

Regarding the functional ability of CD8^+^ T cells found in HESNs, several cohorts of HESNs have been reported to exhibit HIV-specific CD8^+^ T cells secreting IFN-γ and IL-2 (105, 109, 110). These cells also exhibit high activity against the virus, with the production of increased levels of perforin and granzyme B, and both responses were negatively correlated with the time of the last unprotected sexual exposure, suggesting that they could have played a crucial role in avoiding the establishment of the infection (104). Additional reports indicated a broad and strong response of HIV-specific CD8^+^ T cells, characterized mainly by a Tc1 cytokine profile in HESNs when compared with HIV-infected individuals (31, 111).

However, contrasting data exist on the presence of HIV-specific CD8^+^ T cells in HESNs with some studies reporting lack of HIV-specific CD8^+^ T cell responses in these individuals (112); comparisons between HESNs and their HIV seropositive partners showed a lower magnitude of HIV-specific CD8^+^ T cell response and a narrower breadth in HESNs. Nonetheless, the resistance could be related to the recognition of immundominant and conserved viral peptides more than to the recognition of a wider breadth of peptides. Indeed, the peptides recognized by CD8^+^ T cells from HESNs are associated with slow disease progression in cases of HIV infection, and the cytokine profile produced by their HIV-specific CD8^+^ T cells resembled the response observed in HIV-controllers (105, 111). These results suggest that if the HESNs ultimately do acquire the infection, they probably become controllers; however, this remains to be elucidated.

## Conclusion

In summary, the response of CD8^+^ T cells is critical since early stages of HIV infection and its magnitude and effector functions, such as proliferation, cytokine production, and cytotoxic capacity, may determine the control exerted on viral replication and AIDS progression. Furthermore, different subpopulations of these cells in terms of memory and effector phenotype as well as activation level seem to play different roles in viral control. Although these HIV-specific responses have been mainly studied in individuals who achieve a spontaneous and sustained control of the infection, their presence in HESNs suggests that HIV-specific CD8^+^ T cell responses could also play a role in avoiding the acquisition of infection during exposure. Yet, further studies are required to validate this hypothesis.

## Author Contributions

SG, NT, and MR contributed to the design of the review; SG performed the bibliographic search and drafting the initial manuscript. SG, NT, and MR also participated in acquisition and interpretation of data and performing the correction on the work content; approval of the version to be published. Furthermore, these authors are accountable for all aspects of the work in ensuring that questions related to the accuracy or integrity of any part of the work are appropriately investigated and resolved.

## Conflict of Interest Statement

The authors declare that the research was conducted in the absence of any commercial or financial relationships that could be construed as a potential conflict of interest.
